# Converting habits of antibiotic prescribing for respiratory tract infections in German primary care – the cluster-randomized controlled CHANGE-2 trial

**DOI:** 10.1186/1471-2296-13-124

**Published:** 2012-12-20

**Authors:** Attila Altiner, Reinhard Berner, Annette Diener, Gregor Feldmeier, Anna Köchling, Christin Löffler, Helmut Schröder, Achim Siegel, Anja Wollny, Winfried V Kern

**Affiliations:** 1 Institute of General Practice, Rostock University Medical Center, 18055, Rostock, Germany; 2 Centre for Pediatrics and Adolescents, Carl Gustav Carus University Hospital Dresden, 01307, Dresden, Germany; 3 AOK Research Institute (WIdO), 10178, Berlin, Germany; 4 Division of General Practice, Department of Medicine, University Medical Center Freiburg, 79110, Freiburg, Germany; 5 Division of Infectious Diseases, Department of Medicine, University Medical Center Freiburg, 79106, Freiburg, Germany

**Keywords:** Antibiotic prescribing, Respiratory tract infections, Primary care, Randomized controlled trial

## Abstract

**Background:**

With an average prescription rate of 50%, in German primary care antibiotics are still too frequently prescribed for respiratory tract infections. The over-prescription of antibiotics is often explained by perceived patient pressure and fears of a complicated disease progression. The CHANGE-2 trial will test the effectiveness of two interventions to reduce the rate of inappropriate antibiotic prescriptions for adults and children suffering from respiratory tract infections in German primary care.

**Methods/Design:**

The study is a three-arm cluster-randomized controlled trial that measures antibiotic prescription rates over three successive winter periods and reverts to administrative data of the German statutory health insurance company AOK. More than 30,000 patients in two regions of Germany, who visit their general practitioner or pediatrician for respiratory tract infections will be included. Interventions are: A) communication training for general practitioners and pediatricians and B) intervention A plus point-of-care testing. Both interventions are tested against usual care. Outcome measure is the physicians’ antibiotic prescription rate for respiratory tract infections derived from data of the health insurance company AOK. Secondary outcomes include reconsultation rate, complications, and hospital admissions.

**Discussion:**

Major aim of the study is to improve the process of decision-making and to ensure that patients who are likely to benefit from antibiotics are treated accordingly. Our approach is simple to implement and might be used rapidly among general practitioners and pediatricians. We expect the results of this trial to have major impact on antibiotic prescription strategies and practices in Germany, both among general practitioners and pediatricians.

**Trial registration:**

The study is registered at the Current Controlled Trials Ltd (ISRCTN01559032)

## Background

With an average prescription rate of 50%, in German primary care antibiotics are still too frequently prescribed for respiratory tract infections (RTI) [1,2]. Even among children, prescription of antibiotics by general practitioners (GPs) and pediatricians is extensive [3], although it is recognized that antibiotics are very unlikely to alter the course of RTI, such as throat infections, acute otitis media, maxillary sinusitis, and acute bronchitis [4,5]. Consequently, current guidelines advise against the use of antibiotics during the initial treatment of uncomplicated RTI in otherwise healthy children and adults. These guidelines take potential side effects, medicalization for self-limiting conditions, increasing resistance to respiratory pathogens, and costs of unnecessary antibiotic treatment into account [6-9].

Explanations for the inappropriate use of antibiotics in RTIs focus on perceived patient expectations, efforts to rule out a potentially complicated progression of the disease, and inadequate knowledge of physicians [10-14].

Although there is evidence that patients expect antibiotic prescriptions less often than physicians believe [15,16], and that a properly communicated reduction of antibiotic prescriptions does not affect patient satisfaction [17,18], perceived patient pressure, expectations and satisfaction remain major factors influencing the decision whether to prescribe antibiotics or not. During consultations patients often express their worries about symptoms and desire reassurance about the harmlessness of their medical condition. Quite often this perceived pressure leads to unnecessary prescriptions of antibiotics [2,13,15,19-21]. Receiving antibiotics in turn reinforces patients’ beliefs that antibiotics are powerful agents against uncomplicated RTI and strengthens the assumption that future RTI should be treated equally [4,22]. This results in a vicious circle of antibiotic prescription.

Also, diagnostic uncertainty is often mentioned when discussing inappropriate use of antibiotic prescriptions. Studies have shown that clinical indicators such as colored nasal discharge or colored sputum significantly influence prescribing behavior [23]. However, there is only weak evidence that a yellowish or greenish color is a good diagnostic marker for bacterial infection [24,25]. Notwithstanding, GPs still tend to overestimate the likelihood of bacterial infection when evaluating the importance of colored nasal discharge or sputum, leading to increased numbers of antibiotic prescriptions [23].

In the past, numerous trials aimed at optimizing antibiotic prescribing in primary care. Most of them focus on two central problems related to inadequate prescribing: insufficient physician-patient-communication and diagnostic uncertainty. Promising approaches range from feedback on prescriptions and computer-based decision aids [26,27] to communication skills training and point-of-care tests (POCTs) [28,29]. Also, some studies employ the strategy of delayed prescribing. Little and colleagues, for instance, did not prescribe antibiotics in the initial consultation when a viral etiology for acute lower respiratory tract infections was very likely. If, 14 days later, symptoms were not resolved, patients had the opportunity to take a course of antibiotics without reconsultation. This led to a reduction of antibiotic use. Also, patients were less likely to believe in the effectiveness of antibiotics [18].

POCTs are useful to increase diagnostic certainty and help to predict the likelihood of serious bacterial RTI such as pneumonia. POCTs measure inflammatory marker proteins (C-reactive protein [CRP] or procalcitonin). These can be combined with clinical parameters. Other POCTs are fast pathogen identification tests such as rapid streptococcal A antigen detection test (RADT) that indicate benefit from treatment with penicillin [8]. A recent evaluation of an RADT documented a specificity of 98% and a sensitivity of 70%. The latter reached up to 85% when a clinical scoring system (McIsaac score) was implemented to enhance pretest probability [30].

As mentioned above, in a number of recent studies communication training proved to be successful [1,31,32]. In a previous study, we were able to demonstrate a sustained effect of 40% relative reduction of antibiotic prescription for acute cough by motivating GPs to change their doctor-patient communication and by empowering patients [1]. In the Netherlands, Cals et al. conducted a cluster-randomized trial and were able to show that a training in communication skills or POCT for CRP and a combination of both significantly reduced antibiotic prescribing for RTI. Interestingly, these results were obtained without negative impact on patients’ recovery or patient satisfaction [31]. After termination of the trial those physicians who had experience with both interventions were asked about their preferences to manage lower respiratory tract infections. The majority preferred communication skills training to CRP measurement. However, they recognized CRP testing as a useful additional tool to improve diagnostic certainty [33]. Thus, POCTs might be used as an “add on” to physician communication training in order to decrease unnecessary antibiotic prescribing for acute cough.

Based on these findings, the CHANGE-2 trial will test the effectiveness of two interventions aiming at the reduction of inappropriate antibiotic prescriptions for adults and children suffering from RTI in primary care. Intervention A includes communication training. Intervention A+B combines communication training with point-of-care testing. Care as usual serves as control. This is the first trial assessing the effectiveness of the described interventions in a systematic way in Germany. Our primary aim is to improve the decision-making process, but not to reduce antibiotic prescriptions at all costs. This approach ensures that patients who may benefit from antibiotics will be treated accordingly. The trial design allows a comprehensive patient follow-up ensuring that adverse effects of the intended reduction in antibiotic prescription (e.g. hospital admissions) can be monitored. Also, the follow-up will allow for uncovering patient migration and to compare reconsultation rates.

## Methods/design

### Design

The CHANGE-2 trial is a three arm cluster-randomized controlled trial that measures antibiotic prescription rates over three successive winter periods and reverts to administrative data of the German statutory health insurance company AOK.

### Intervention

Clusters will consist of participating primary care physicians who will be randomized into three groups: Intervention A (communication training), intervention A+B (communication training + POCT), and control. Communication training will be organized within one-time small group sessions and will focus on the following topics: Patient expectations, shared decision-making (SDM), and the concept and use of delayed prescribing. In particular, participating physicians will ameliorate their communication techniques in order to explore patients’ (or parents’) expectations. Also, they will be trained in patients’ concepts of disease and patients’ actual needs, e.g. ruling out a serious disease or pain relief [34,35]. This concept incorporates the principles of shared decision-making, without weakening the role of the primary care physician [36]. Furthermore, an adapted concept of delayed prescribing will be presented to participating physicians. This concept might be useful for those (rare) cases in which – despite communication training – the issue of antibiotic prescription cannot be solved in a satisfactory manner.

Physicians randomized into intervention A+B will be encouraged to use POCT kits (CRP and RADT) when appropriate. These will be provided free of charge. Physicians and their practice staff will be trained on how to use these test kits and how to judge which patients might benefit from POCTs. Among intervention A+B we expect up to 15% of patients to be tested with RADT, and up to 40% of patients to be tested with CRP.

### Randomization

The study is carried out in primary health care facilities of the two German regions of Baden-Württemberg and Mecklenburg-Western Pomerania. Clusters will consist of general practitioners or practice-based pediatricians and their patients, who ask for consultation due to acute respiratory infection. Using complete and current lists of general practitioners and practice-based pediatricians provided by the Associations of Statutory Health Insurance Physicians of both regions potential participants will be contacted. Interested GPs and pediatricians will be cluster-randomized to intervention A, A+B or control.

### Study population and recruitment

Patients will be recruited in the practices of participating physicians at three points in time: during a three months period in winter at baseline (T0), one year after baseline after the educational intervention (T1) and two years after baseline (T2). Diagnosis and relevant results of clinical examination will be documented. From previous studies we know that in German primary care during winter season a primary care physician sees on average 20 patients per week who suffer from RTI. This number might increase up to 30–50 patients during peak periods. However, for our calculations we rely on a conservative estimation of 15 patients per week. Within a typical primary care setting of the two considered regional areas, at least 40% of all patients are insured with AOK. As a result, we assume to recruit at least 6 patients per week. Each recruitment period (baseline, T1 and T2) will last 12 work weeks (excluding holidays). Thus, we expect about 70 eligible patients to be included per participating primary care physician. For smaller practices that might face difficulties recruiting this number of patients, we allow for the extension of the recruitment period for up to 8 weeks (thus, 20 weeks in total). The personnel of the participating practices will not be involved in the collection of outcome relevant data.

Inclusion criteria for patients are: health insurance with the AOK, 3 months minimum age, physician consultation visit due to the first episode of acute RTI (upper respiratory tract infection [URTI] and lower respiratory tract infection [LRTI]) according to the ICD classes: J00-J04, J06, J13, J20, J22, otherwise healthy. This definition will include all typical acute RTI including bronchitis, tonsillopharyngitis (e.g. sore throat), and otitis media. Participants are required to give informed consent that includes the acceptance of scientific use of relevant data stored at the AOK.

Patient exclusion criteria are underlying chronic diseases, which may affect the immune status in any relevant matter. This includes malignoma, chronic obstructive pulmonary diseases, cystic fibrosis, and immune deficiency of other causes.

### Aim of the study and outcome measures

**Figure 1:**
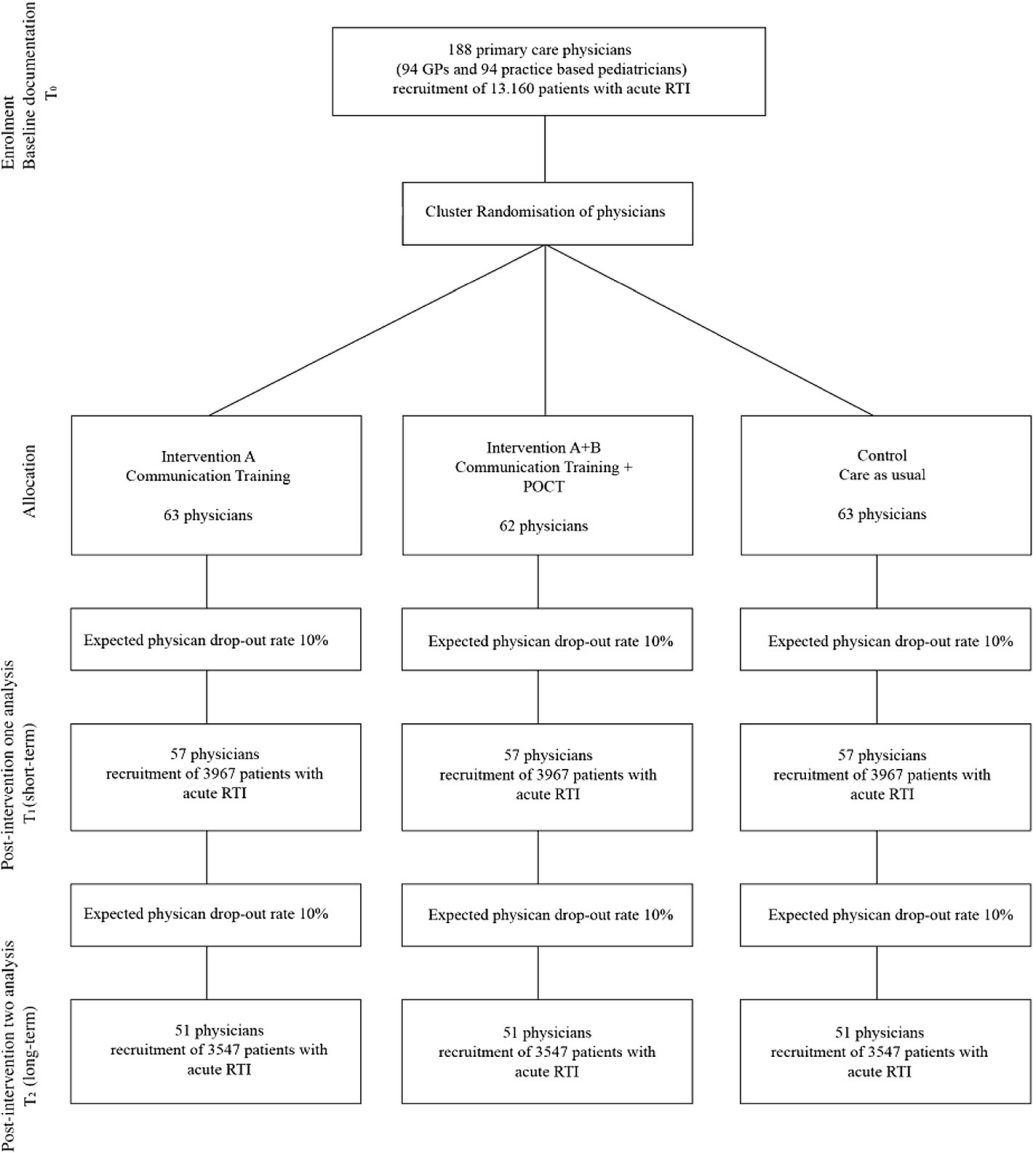
Flow chart of the CHANGE-2 trial.

The trial assesses the effectiveness of two interventional approaches to reduce unnecessary antibiotic prescription in primary care. Primary outcome measure is the physician antibiotic prescription rate for RTI at study period T2 derived from the data of the AOK health insurance company. As secondary outcome measures we will include reconsultation rate, complications/adverse effects (including hospital admissions), choice of guideline conform antibiotic substance in case one was prescribed. Outcomes will be measured at baseline (T0), shortly after the interventions took place (T1) and approximately 12 months after the interventions (T2) (Figure 1).

### Sample size calculation

For power calculations we considered a relative reduction of the overall antibiotic prescriptions of 30% as clinically relevant and realistic. Our prior study proved that by means of specific communication training among primary care practices a relative reduction of even 40% in antibiotic prescribing is possible [1]. If a reduction of a prescription rate from 50% to 35% (relative reduction of 30%) was to be demonstrated with a power of 80% at a significance level of 1.67% two-sided, a sample size of 240 per group would be required in a randomized trial.

The results from our prior study allow us to estimate the intraclass correlation coefficient (ICC) with high accuracy for the sample size calculation. Assuming an intraclass correlation of 0.2 and a cluster size of 70 patients [1], this sample size has to be multiplied with a design factor of 14.8, resulting in a sample size of 10,656 evaluable patients in 152 practices for a 3-arm-trial with pairwise comparisons. With a practice drop-out rate from T0 to T1 of 10% and again of 10% from T1 to T2, a total sample size of 13,160 patients in 188 practices has to be recruited for the study at baseline to ensure that at T2 152 practice-based physicians with 10,640 patients can be analyzed.

### Statistical analyses

For the primary endpoint a generalized multi-level model, that takes the randomized clusters (practices) as random effect into account, with antibiotic prescription rate at T2 as dependent and random group as independent variable, will be fitted to the data. Baseline (T0) antibiotic rates and a selection of further baseline characteristics of physician or patient will function as covariates. Points to consider on adjustment for baseline covariates on patient level will be amongst others severity of illness on a 4-point scale, fever, patient smoking or not, duration of symptoms before seeing the doctor and patient’s age [37]. The intervention effects are quantified by the between-groups odds ratios of the corresponding estimates of changes from baseline from the fully adjusted model, which we assume to give the best account of the study results. The primary analysis will consist of the three pairwise comparisons between study arms (comparisons to control for efficacy proof, between active arms for comparative effectiveness), each at a test level of 1.67% to keep an overall level of 5% for the total primary analysis. The short-term assessment of the primary endpoint at T1 and all secondary endpoints at T1 or T2 will be analyzed using analogous models.

### Methods against bias and data quality

Selection bias will be minimized by a standardized and scrupulously followed recruitment procedure, supervised by research assistants and monitored by the Clinical Trial Center North of the University Hospital Hamburg-Eppendorf (CTCN). The personnel of the participating practices will not be involved in the collection of outcome relevant data. Follow-up data will be obtained directly by study centers. Clusters are randomized to treatments to avoid selection bias. Practices and patient recruitment will be closely monitored and regular practice visits will ensure good collaboration. Both participating physicians and their practice staff will be compensated financially. Practice efforts for recruitment and basis documentation procedures and tools (forms) will be optimized for excellent usability.

Before start, the study was registered in a public Internet trial archive (Current Controlled Trials Ltd, ISRCTN01559032). The Clinical Trial Center North and the University Hospital Hamburg-Eppendorf will monitor the study. Good Clinical Practice guidelines and CTCN standard operation procedures will be followed.

### Ethical approval

The protocol was approved by the ethics committee of the Rostock University Medical Center before recruitment of physicians and patients on September 10^th^ 2012 (A 2012–0108).

Patients will have to sign an informed consent form prior to enrolment in the study. It is not expected that participation in the study will expose the patients to relevant risks. GPs, pediatricians, and patients will participate voluntarily and will be allowed to abandon their participation at any time and without giving reasons.

## Discussion

The previously conducted CHANGE trial provides evidence for the effectiveness of doctor-patient communication and patient empowerment for reducing antibiotic prescriptions in German primary care. CHANGE-2 aims at investigating the additional benefit of point-of-care tests in this setting. Earlier research found positive effects in the Netherlands [28,33]. As both countries have a similar antibiotic prescription pattern, that is comparatively low rates [38], we assume a similar effect in Germany.

In fact, a recent international study analyzed the effect of a multifaceted intervention that included training for appropriate use of antibiotics, posters and brochures for patients, and access to POCTs. Communication training was not included here. Whereas antibiotic prescription rates were markedly reduced in countries with initially high rates, no significant effects were found in low prescribing countries [39]. This finding suggests that distribution of information alone is not sufficient to further reduce prescription rates in low prescription countries.

Both proposed interventions (communication training with and without POCT) do not aim at reducing antibiotic prescriptions at all costs, but on improving the process of decision-making. This approach will ensure that patients who might benefit from antibiotics will be treated accordingly. In light of the current German average prescription rate of 50% there is significant room for improvement: an antibiotic prescribing level of 10–15% for acute RTI is in fact reasonable [40,41].

As the outcomes will be analyzed based on the data of the AOK sickness fund, a comprehensive patient follow-up is possible and ensures that adverse effects (e.g. hospital admissions) can be monitored. Furthermore, the follow-up will uncover patient migration, allowing for the comparison of reconsultation rates. Since there is no evidence on the interrelationship between reduced antibiotic prescriptions and severe complications due to bacterial infections, we particularly focus on this issue. If data analysis will show a significant difference in hospital admissions due to reduced antibiotic prescriptions, the trial will be stopped. In addition, we will analyze all spontaneous reports of adverse effects.

As far as limitations are concerned, there are potential sources of selection bias: Firstly, physicians volunteering to participate in our study might be likely to be those most interested in and sensitized for the issue of antibiotic prescriptions. These physicians might be more likely to change their prescription behavior than other physicians. Secondly, as we will only include patients insured with the health insurance AOK, a bias on the patient level might be caused.

This trial will be the first randomized controlled trial in Germany to evaluate the use of communication training and POCTs on antibiotic prescription rates for RTI. We expect the results of this trial to have major impact on antibiotic prescription strategies and practices in Germany, both among GPs as well as pediatricians. The approach is simple to implement and might be used very rapidly among the target group. This study will also allow gaining more insights into the natural course of RTI, a common but still under-researched illness. Provided that our intervention proves to be successful on a large scale, we expect our findings to disseminate rapidly among regional and national health insurances, professional societies, and networks.

## Abbreviations

AOK: Allgemeine Ortskrankenkasse (German statutory health insurance company); CRP: C-reactive protein; CTCN: Clinical Trial Center North; GP: General practitioner; ICC: Intraclass correlation coefficient; ICD: International Statistical Classification of Diseases and Related Health Problems; LRTI: Lower respiratory tract infection; POCT: Point-of-care test; RADT: Rapid streptococcal A antigen detection test; RTI: Respiratory tract infection; URTI: Upper respiratory tract infection.

## Competing interests

The authors declare that they have no competing interests.

## Authors’ contributions

AA, WVK and RB initiated and designed the study; all authors performed further development. The paper was drafted by AA, CL and AD and all authors read and approved the final manuscript.

## Pre-publication history

The pre-publication history for this paper can be accessed here:

http://www.biomedcentral.com/1471-2296/13/124/prepub
